# High quality standards for a large-scale prospective population-based observational cohort: Constances

**DOI:** 10.1186/s12889-016-3439-5

**Published:** 2016-08-25

**Authors:** Fabrice Ruiz, Marcel Goldberg, Sylvie Lemonnier, Anna Ozguler, Evelyne Boos, Alain Brigand, Violaine Giraud, Thierry Perez, Nicolas Roche, Marie Zins

**Affiliations:** 1 CLINSEARCH, 110 Avenue Pierre Brossolette, 92240 Malakoff, France; 2 UMS 011 Inserm - UVSQ « Cohortes épidémiologiques en population », 16 avenue Paul Vaillant Couturier, 94 807 Villejuif, France; 3 Centre d’examens de santé d’Angoulême, 5 rue la Croix Lanauve, 16024 Angoulême, France; 4 AP-HP, Service de Pneumologie et d’Oncologie Thoracique, Hôpital Ambroise-Paré, Boulogne, France; 5 Service de Pneumologie et Service EFR, Hôpital Calmette, CHU Lille, F-59000 Lille, France; 6 EA2511, Université Paris Descartes et Service de Pneumologie, Hôpital Cochin, 27 rue du Fg Saint Jacques, 75014 Paris, France

**Keywords:** Cohort study, Epidemiological methods, Measurement tool development, Methodology, Respiratory

## Abstract

**Background:**

Long-term multicentre studies are subject to numerous factors that may affect the integrity of their conclusions. Quality control and standardization of data collection are crucial to minimise the biases induced by these factors. Nevertheless, tools implemented to manage biases are rarely described in publications about population-based cohorts. This report aims to describe the processes implemented to control biases in the Constances cohort taking lung function results as an example.

**Methods:**

Constances is a general-purpose population-based cohort of 200,000 participants. Volunteers attend physical examinations at baseline and then every 5 years at selected study sites. Medical device specifications and measurement methods have to comply with Standard Operating Procedures developed by experts. Protocol deviations are assessed by on-site inspections and database controls. In February 2016, more than 94,000 participants yielding around 30 million readings from physical exams, had been covered by our quality program.

**Results:**

Participating centres accepted to revise their practices in accordance with the study research specifications. Distributors of medical devices were asked to comply with international guidelines and Constances requirements. Close monitoring enhanced the quality of measurements and recordings of the physical exams. Regarding lung function testing, spirometry acceptability rates per operator doubled in some sites within a few months and global repeatability reached 96.7 % for 29,772 acceptable maneuvers.

**Conclusions:**

Despite Constances volunteers being followed in multiple sites with heterogeneous materials, the investment of significant resources to set up and maintain a continuous quality management process has proved effective in preventing drifts and improving accuracy of collected data.

## Background

Developing large-scale prospective cohorts is of utmost importance to address epidemiological, clinical or pathophysiological research questions. In such cohorts, quality control is a major issue to ensure that collected data are sufficiently reliable and robust.

Constances is a large population-based general-purpose cohort intended to serve as an open epidemiologic research infrastructure accessible to the research community and to provide public health information [1]. At term, it will comprise a sample of 200,000 participants aged 18–69 years at inclusion who are representative of the French adult population. The survey was launched towards the end of 2012 and has already included more than 94,000 volunteers (February 2016). New participants are continuously being included and the cohort will be fully constituted by the end of 2018.

**Fig. 1:**
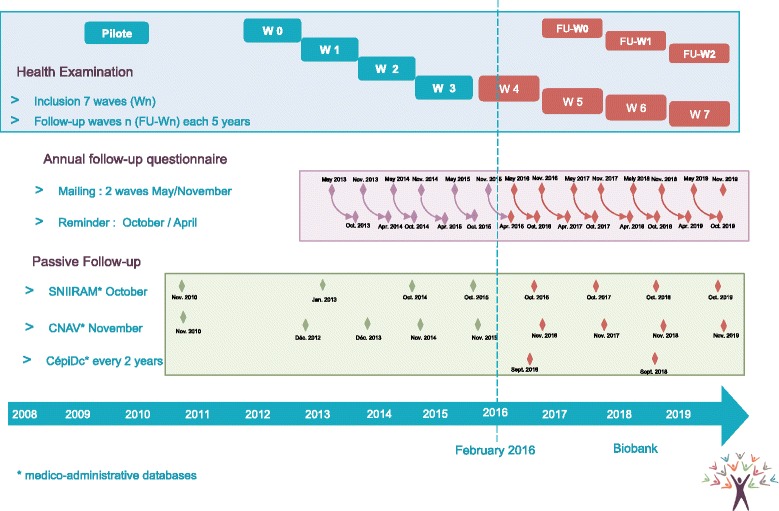
Study progression

At inclusion, volunteers are asked to complete a questionnaire and attend a medical exam in one of the 20 participating recruitment sites located throughout France. These sites are Health Screening Centres (HSC) managed by the national social security fund (CNAMTS) to provide free health check-ups for the French population [2]. As well as the medical examination, laboratory tests and questionnaires, data related to the participants are extracted from national health and social databases on a regular basis. The volunteers are followed up by annual questionnaires and invited to attend a new medical exam at a participating site every 5 years (Fig. 1).

During the physical exam, biometry (weight, height, waist and hip circumference), blood pressure, electrocardiogram, vision, hearing and lung function are measured. Blood and urine samples are collected for laboratory tests. Cognitive and physical functioning assessments are conducted for volunteers 45 years and older.

All the data collected in the HSCs as well as from the other data sources (questionnaires, administrative databases) are transferred to the central Constances site where the data are consolidated in a single centralized database.

Numerous procedures and controls have been set up for the Constances study in order to ensure that the data is of high quality and reproducible and to allow appropriate assessment of potential measurement biases. The present paper focuses on quality issues encountered in the cohort, describes how these issues were addressed and the results of corrective measures with special interest in spirometry as an illustrative example. For clinical chemistry, several publications demonstrate the importance of close monitoring to correctly assess these biases. A recent example from Withehall II emphasized this necessity for cognitive testing [3]; non-adherence to the protocol was not identified at the time of the data collection leading to complex identification of outliers.

However, it would have been very ambitious to cover all parts of the protocol in a single article. We therefore intend to discuss the biological and cognitive parts in separate papers. We describe here the quality assurance and controls implemented for the medical examination part of the protocol.

## Methods

### Quality assurance

#### Standard operating procedures

One goal of the study protocol and Standard Operating Procedures (SOPs, which define the medical device specifications and detail the measurement methods for each type of data) was to standardize methods to ensure successful replication of data collection for all volunteers irrespective of when, where and by whom they are performed [4, 5].

For that purpose, we organized working groups supervised by experts from each domain and composed of personnel from the participating sites (MDs and nurses), experts of the corresponding field, epidemiologists and quality assurance specialists.

The SOPs describe the materiel admissible for the study, the required annual certification, and the periodic checks or maintenance. All the steps of each measurement are detailed to minimize inter-operator variability [5] Table 1.

**Table 1:** Required equipment and maintenance rules

Exam	Equipment recommendations	Daily verification	Monthly verification	Annual verification	Procedure
Weight	• Class III medical scale CE marked • Required precision: ± 0.1 kg • Amplitude: 2.0–150.0 kg minimum	• Spirit level verification • Zero displayed before use	NA	• Performed by an external certified body.	• Volunteer in underwear, motionless. • Measurement read by the nurse once stabilization obtained.
Height	• Measuring rod • Graduation: 1 cm • Amplitude: 60–200 cm • 10 cm space between heels indicated by floor markings.	• 0 graduation at the ground level – 1 mm tolerance permitted	• Cross-checking with the tape measure	• Performed by an external certified body.	• Volunteer standing, arms at sides, inside heels 10 cm apart, head upright, looking straight, deep breath in. Measurement read by the nurse, with slider at eye-level.
Waist-hips-abdominal circumference	• Tape measure with 2 sides of different colors. • Graduation: 1 cm • Amplitude: 0–150 cm	• Signs of wear	• Cross-checking with the measuring rod	• Renew every year	• Volunteer in underwear, standing with feet shoulder-width apart, arms slightly apart, motionless. • Palpation of anatomical landmarks for right positioning (Waist: mid-axillary line; Hips: widest level over the greater trochanters) • Ensure horizontality of the tape measure with no twists. • Tape measure snug but not digging into the skin. • Measurement read by the nurse on the exhale.
Arterial pressure	• Tensiometers provided by The “Constances” research team: OMRON 705. • Accuracy of measurement: ±3 mmHg • Amplitude: 0–300 mmHg	• Search for anomalies of the entire pneumatic circuit	NA	• Centralized and organized by the Constances research team	• Point of reference on the blood pressure cuff according to the arm circumference. • Volunteer in supine position. • First reading after a 5-minute rest, second reading on the contralateral arm, third reading after a 1-minute pause on the arm with the highest systolic blood pressure. • Orthostatic hypotension measured after standing for 1 min
ECG	• 12 standard ECG leads • Screen display and software program that ensures the overall management • Recording features: speed (25 mm per second), amplitude (10 mm by mV), leads and filters. • Examination table with a minimum width of 65 cm to avoid muscular contractions or arms falling off table.	• Signs of wear. • Calibration signal at the beginning of each trace.	NA	• Performed by an external certified body.	• Volunteer in supine position, calm, arms at sides. • Thorough skin preparation (remove grease) • Ensure a good electrode-to-patient contact. • Palpation of anatomical landmarks for proper placement of the electrodes
Far visual acuity	• Monoyer scale • Floor marking to indicate the required reading distance of 3 or 5 m • Unit: 1/10 • Required precision: ± 1/10 • Amplitude: 0/10–10/10	• Signs of wear, dirt	• Signs of wear	NA	• Volunteer standing or seated • For monocular visual acuity: cover placed on one eye with no pressure
Near visual acuity	• Parinaud scale • Unit: Parinaud • Amplitude: 1.5–20 • Specific device provided by Constances to standardize the scale-brow distance (33 cm).	• Signs of wear, dirt	• Signs of wear	NA	• Volunteer seated • Specific device in contact with the volunteer’s brow • For monocular visual acuity: cover placed on one eye with no pressure
Audition	• Required precision: ± 3 dB from 500 to 4,000Hz and ± 5 dB beyond • Amplitude: −10 to 85 dB by 5 dB steps minimum • Test hearing by air conduction using a pulsed tone at various frequencies (from −10 to 85 dB – change by 5 dB steps).	• Control test of each frequency at 60 dB both sides	NA	• Performed by an external certified body	• Response mode: hand raised / response button pressed, held as long as tone is heard • Volunteer unable to see the operator • Headphones placed by the operator • Start by a demonstration • Otoscopy (to detect excessive earwax)
Spirometry	• As per requested by ATS/ERS task force • Unit: L • Required precision: 0.035 L • Amplitude: 0–8 L	• Performed in accordance with the ATS/ERS guidelines	NA	• Performed by an external certified body in accordance with the ATS/ERS guidelines	• Performed in accordance with the ATS/ERS guidelines

### Material

The medical devices with their specific software and the middleware are purchased by the HSCs at a local level. Consequently, not all the participating sites have the same equipment. As precision and accuracy could differ depending on the material used, the manufacturer’s instructions for each device used for the measurements were initially reviewed [6] and are updated throughout the study to take into account any changes in material or versions of middleware.

If the technology of a device undergoes significant modifications, the measurements for a series of volunteers are doubled within a short period (first with the new device and second with the previous one).

Quality controls performed during the pilot study revealed that various middleware used by the study nurses did not transfer the exact value of the medical device measurements to the local database. These discrepancies were reported to the corresponding study site or to the medical device distributor and they were encouraged to quickly update their software. The correct values were added to the study Case Report Form (CRF) manually.

### Pre-study site qualification

Prior to inclusion, we performed an on-site inspection of each HSC and presented the study to the staff. During this visit, we checked the staff’s qualifications, the study-related equipment in the patient examination rooms and the source documentation. We also obtained the investigators’ agreements to allow the site monitors direct access to the relevant data sources.

### Training

All study site personnel involved in the study had to be trained by a study monitor or an experienced member of the site staff prior to participation. Training certificates were delivered by the monitor, stored on site, and registered in a monitoring database. A study monitor was also present on site for the first 2 days of inclusion to support the study staff.

### Monitoring of measurements

To minimize drifts over time [7], the practices of all the study-site staff are monitored on a monthly basis. All steps of each SOP are recorded on a detailed checklist and the monitor ensures that the observed practices are compliant with the protocol and SOPs. Deviations are documented in a visit report and the related standards are reviewed with the study site member.

### Case report form and data transfer

All the medical records of the participating sites are electronic and data are stored in a local database, the structure of which is common to all sites. Most of the data collected during the physical examinations are then extracted from the local database and exported to the Constances data centre with cryptography procedures. This is to ensure standardization of a part of the data collection and to reduce potential data capture errors by the study nurse who enters source document information in the study CRF [8].

For research purposes, the experts requested to increase the precision of some data and to collect some additional specific items. To satisfy their expectations a CRF was specifically designed to complete the data collection.

## Quality control process

### Validation plan

Two validation plans run permanently in the database. The first consists in a crosscheck of identical data collected from different sources, the data entered in the CRF being compared with the data extracted from the electronic medical records of the sites to check for consistency. This is feasible when the study procedures are requesting, for a specific item, a higher precision than the value usually entered in the medical records of the sites. The second validation plan tracks missing data, discrepancies between different questionnaires, out of range values or any warning requiring consistency check as predefined by the researchers.

Data flagged by the validation plans generate queries processed directly by the data managers or forwarded to the monitors to be answered during the on-site monitoring visits.

### On-site verification

Each month samples of the data exported from the site and imported in the Constances database are extracted from the database for quality controls. First, for each category of data the monitor identifies the source document (where the data was initially recorded). For data entered directly in the local electronic medical record, the site prints out the results. The study monitor then checks the consistency between the site data and the Constances database extraction.

For any discrepant data, the study monitor identifies the origin of the discrepancy. In the case of data entry error, the correction is transmitted to the Constances data centre. If the discrepancy does not originate from the data entry process, then one of the electronic data transfers may be involved (medical device to middleware, middleware to electronic medical records, or electronic medical records to the Constances datacentre). In this case, the monitor must identify the systematic error and all related data have to be corrected and reintegrated into the Constances database.

### Permanent monitoring of the inter-operator and inter-site variability

Inter-operator variability is an important indicator of quality [9]. It is also an important tool for monitoring practices, identifying drifts and generating targeted reminders and training. Although biometric measurements are routinely tested, we also decided to follow-up other variables as indicators of SOP compliance. Examples are shown in Table 2.

**Table 2:** Indicators of SOP compliance

Measurement	Indicator	Error
Weight	Distribution of decimal	Results rounded by operator
Waist	(Waist/Abdominal) ratio	Palpation of anatomical landmarks not systematically performed by operator
Arterial pressure	Distribution of the left/right side for the 3^rd^ reading	Two first readings performed on the same arm
Arterial pressure	Higher blood pressure	Chronograph not respected
Near visual activity	Lower Parinaud	Specific device to respect distance not used
Far visual activity	Higher scores in 1/10	Distance not respected
Audition	High distribution of last digit results equal to 0	Test performed with steps of 10db instead of 5 dB
Audition	Lower percentage of patient with negative scores	Test performed without testing negative values.
Spirometry	Lower percentage of acceptable maneuver declared	Insufficient patient coaching or training of the study-nurse
Spirometry	(repeatability declared/repeatability calculated) ratio	Insufficient training of the study nurse

## Results

### An example of the quality assurance and control in Constances: lung function testing

With more than 94,000 participants included by February 2016, around 30 million readings from physical exams have been covered by our quality policy system. To discuss the issues and the first results, we decided to take the example of spirometry, one of the eight measurements performed.

The whole process of lung function testing is detailed in depth in the ATS/ERS guidelines [10]. The study monitor checks that the study-nurse adheres to all the requirements during the on-site visits. Within- and between-maneuver quality criteria are checked on site by the monitor and reproducibility is systematically assessed for all subjects as part of the validation plan program.

While most of the requirements are objective and can thus be checked without a shadow of doubt, one of the ARS/ERS guidelines criterion is more subjective: categorizing the maneuver as acceptable after inspection of the flow-volume curve. As this involves some interpretation, the Respiratory Experts Review Board organizes an annual central review to assess the curves considered as acceptable from 1 % of the curves randomly sampled each month.

### Material and distributors

During the pre-study site qualification phase, we studied the device documentation collected from each site (technical notice, operating manual and commercial leaflet) to identify differences between the operator practices and our SOP.

Although all the manufacturers equipping the Constances sites produced documentation stating that their devices satisfy the ATS/ERS recommendations, some concerns arose from analyzing practices and the device results sheets.

As some of the instructions provided by the distributors were actually not in accordance with ATS/ERS recommendations, we experienced difficulties explaining to the study nurses that they would have to modify their practice. The strongest resistance we met was to re-introduce daily quality control in sites equipped with ultrasound spirometers. The distributor’s documentation states that the ultrasound spirometers require neither calibration nor calibration checks. Calibration establishes the relationship between sensor-determined values of flow or volume and the actual flow or volume, whereas calibration checks validate that the device is within calibration limits [10]. With pneumotachographs (the spirometers used by the sites before acquiring an ultrasound device), calibration and calibration checks were closely linked.

Distributors promote ultrasound spirometers by highlighting that they no longer require any form of calibration and even suggest that the calibration syringe would become redundant. Once equipped with ultrasound spirometers, the sites followed this advice and stopped performing the calibration procedures. When we reminded the sites that ATS/ERS guidelines state that it is mandatory to perform daily verification, the distributor used a published study to assert that verification was not necessary [11]. The abstract of this study, claiming a volume accuracy higher than 3 % for at least 4 years, encouraged the users of the referenced ultrasound spirometer to abandon daily calibration checks.

To convince the sites to restore daily calibration checks, we used arguments extracted from the same publication in its entirety. Indeed, the authors explained in the methods section that, as recalibration of the device was not possible, users repeated verification until success. Failures were not logged and analysis was limited to the range of the successful verifications. The authors minimized this bias and explained in the limitations section that the initial failures were almost always due to human error. The authors revealed an additional important fact: two of the 34 spirometers had been returned to the manufacturer after repeated verification failures.

After having considered these study results, the sites became sensitive to the importance of daily verification, understanding that these devices could also malfunction and that the best way of preventing the generation of corrupted data remained daily verifications.

In some sites using pneumotachographs, we also identified unacceptable communication from some distributors. When some sites experienced difficulties calibrating the spirometer, one distributor offered to redefine some parameters, which consisted in actually changing the acceptable error from 3 to 10 %. The sites refused to have the parameters modified and did not perform the exam until the manufacturer had replaced the devices.

Other issues were also identified. For example, the results sheets for some spirometers mentioned “ERS 1993” [12]. The distributor confirmed that the criteria had not been updated in order to obtain more repeatable maneuvers (difference of <0.2 l versus <0.15 l according to the 2005 criteria).

With most brands, we also identified errors in the decision tree programs for Forced Vital Capacity (FVC) and Forced Expiratory Volume in 1 s (FEV_1_) recordings. We noticed that after having obtained three acceptable and two repeatable maneuvers, FVC and FEV_1_ could be extracted from the same maneuver whereas the largest FVC and the largest FEV_1_ did not belong to the same curve. As ATS/ERS guidelines recommend that “the largest FVC and the largest FEV_1_ (BTPS) should be recorded […] even if they do not come from the same curve”, we reported this issue to the distributors or manufacturers. As they were not reactive, we had to change the Case Report Form to collect FVC and FEV_1_ of the three usable curves.

### Operator follow-up and training

Even though operators had been performing spirometry in their daily practice for years, training was provided by the study monitor during the “site initiation visit”. Monthly on-site verification by study monitors found that some parts of the SOP were not implemented, such as hourly temperature control or demonstration of the technique to the patient. Once the study monitor had identified a deviation, he/she systematically retrained the operator. However, the presence of a study monitor in the examination room inevitably makes the operator more vigilant about potential protocol deviations. To prevent this evaluation bias, we analyzed the rate of acceptable maneuvers declared per site and per operator for the first 6329 spirograms collected. We noted considerable differences between sites: while the overall average percentage of acceptable maneuvers was 69 % (4369 of 6329), they ranged from 35.2 % (135 of 384) to 91.5 % (108 of 118).

The site with the lowest performance had conducted 384 spirograms, involving 13 different operators who had conducted more than ten exams for the study; the percentage of acceptable spirograms per operator varied from 11 to 65 %. However, as non-acceptable maneuvers were not sent to the Constances data centre, the weakness of some operators did not impact the global quality of FVC and FEV_1_ values collected.

The repeatability of the acceptable maneuvers (according to the 2005 criteria) is also an important indicator of quality. This indicator was high-grade: 93.4 % of the acceptable maneuvers were declared repeatable (4081 of 4369) ranging from 82.1 to 100 % according to the site. The repeatability of each maneuver was checked by calculation: although no more than 1.1 % (45 of 4081) were wrongly declared as repeatable, 277 were incorrectly declared as non-repeatable, giving a total of 97.5 % (4260 of 4369) repeatable spirograms. These global rates were consistent with the best data reported in the international literature [13, 14].

**Fig. 2:**
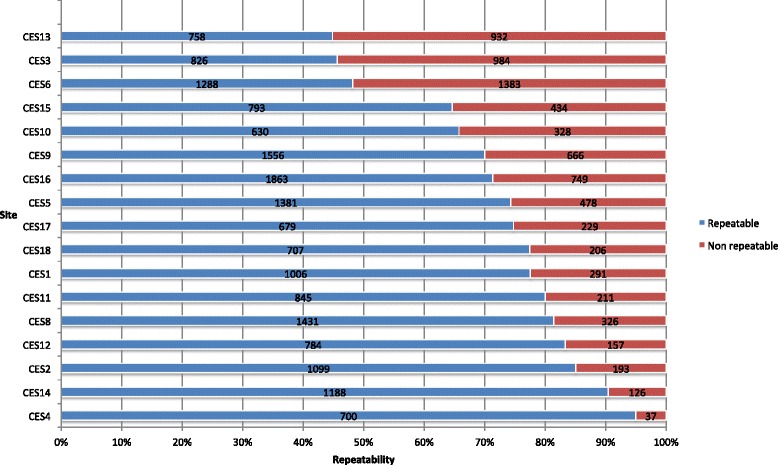
Graph: “Repeatable maneuvers per site end 2013”. X-axis: “% of repeatable maneuvers”. Y-axis: “Site”. “Repeatability calculated from results of 25 848 maneuvers”. *Blue* “Repeatable”. *Red* “Non repeatable”

We thus concluded that the factor that we had to improve was the acceptability rates for some operators. Thus, the sites were sent the acceptability and repeatability rates per operator and per site compared to the other sites. We subsequently contacted the sites to discuss these figures. Most were very reactive and organized additional training. By the end of 2013, all the sites had increased their acceptability and repeatability rates. The percentage of acceptable and repeatable maneuvers (calculated for all maneuvers performed) ranged from 44.9 % (758 of 1690) to 95.0 % (700 of 737). Figure 2 presents the inter-site variability of these rates.

**Fig. 3:**
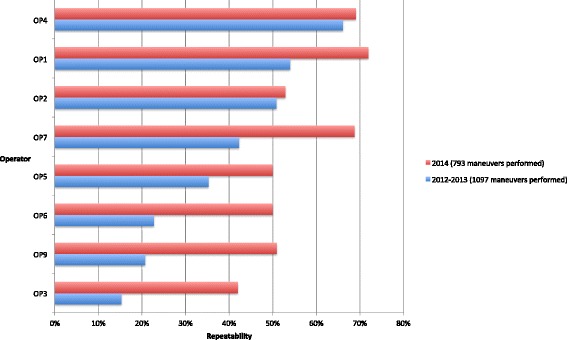
Graph: “CES 13: Repeatable maneuvers per operator”. X-axis: “% of repeatable maneuvers”. Y-axis: “Operator”. “Repeatability calculated for operators having performed >100 maneuvers during the first period”. *Blue*: “2012–2013 (1 097 maneuvers performed)”. *Red*: “2014 (793 maneuvers performed)

Some sites requested individual status reports to follow the progression of each operator, and we noticed that the percentage of acceptable spirograms increased for each of them. Figure 3 illustrates the quality improvement of repeatability per operator in the weakest site 1 year after corrective actions had been introduced.

### Central review verification

In August 2014, the Respiratory Experts Review Board received a random sample of maneuvers (20 per site) qualified by the sites as acceptable and repeatable. A total of 335 maneuvers were subsequently examined by the Board.

They considered that 99.4 % of the maneuvers were free from artefacts and that 98.2 % had a good start. Unexpectedly, they noticed that the exhalation duration was not provided in the results report in most cases (85.9 %) but that volume-time curves showed an acceptable plateau. Hence the Board decided to ask the study monitors to measure the exhalation duration of maneuvers in one or two volunteers during each of their monthly on-site visits so as to record this value for approximately 1 % of the participants.

For between-maneuver criteria, the Respiratory Experts Review Board was not able to evaluate the repeatability criteria for 26.6 % of the maneuvers as only the greatest FVC and FEV_1_ values of the same curve (considered by the spirometer software as the best one) were printed out. They reported that 94.6 % of the 239 remaining maneuvers were repeatable, which is consistent with our analysis of a larger sample (Table 3). We could verify that 96.7 % of the maneuvers satisfied the between-maneuver criteria.

**Table 3:** Acceptable maneuvers reported by the sites (*N* = 29,799)

Repeatability criteria satisfied			
Study nurse assessment reported in the CRF	Calculated with FVC,FEV_1_ data entered in the CRF	Frequency	Percentage
Repeatable	Repeatable	28,396	95.36
Not repeatable	Repeatable	253	0.85
Assessment missing	Repeatable	151	0.51
Repeatable	Not repeatable	804	2.70
Not repeatable	Not repeatable	168	0.56
Assessment missing	Not repeatable	7	0.02

## Discussion

The collection of high quality data for 200,000 participants attending a physical examination, and undergoing laboratory tests and cognitive and functional tests relies on active participation of all participating centres. As described for lung function testing, the study-site personnel accepted to adapt their practices to the study research specifications for all exams in the interests of standardizing the measurements and the study report documentation. In parallel, the medical device distributors were challenged to satisfy Constances requirements. Albeit not perfect, the Constances the quality assurance and control program showed to be efficient.

## Conclusions

The study team invests considerable human and financial resources to implement and maintain a high quality management process to produce data by rigorous control of materials and methods. Similar improvements as those described for the spirometry have also been observed for the other physical examinations. All in all, researchers will be able to use the data generated from Constances confident in the knowledge that the data were collected via robust processes.

## Abbreviations

ATS, American Thoracic Society; BTPS, body temperature and pressure, saturated; CNAMTS, Caisse nationale d’assurance maladie des travailleurs salariés; CRF, case report form; ERS, European Respiratory Society; FEV_1_, forced expiratory volume in 1 s; FVC, forced vital capacity; NA, not applicable; SOP, standard operating procedures
